# Supplementary material for: Gaussian synapses for probabilistic neural networks
Source: Nat Commun. 2019 Sep 13;10:4199. doi: 10.1038/s41467-019-12035-6 (PMC6744503; doi:10.1038/s41467-019-12035-6)
Supplement: Supplementary file 1 — Supplementary Information [file 41467_2019_12035_MOESM1_ESM.pdf]

Supplementary Information

Gaussian Synapses for Probabilistic Neural Network

Amritanand Sebastian[1], Andrew Pannone[1], Shiva Subbulakshmi Radhakrishnan[1, 2], and Saptarshi Das[1,3,4,]*

[1]*Department of Engineering Science and Mechanics, Pennsylvania State University, University Park, PA 16802, USA*

[2] *Electrical and Electronics Engineering, Amrita Vishwa Vidyapeetham, Ettimadai, Coimbatore, Tamil Nadu 641112, India*

[3]*Department of Material Science and Engineering, Pennsylvania State University, University Park, PA 16802, USA*

[4]*Materials Research Institute, Pennsylvania State University, University Park, PA 16802, USA*

Supplementary Note 1: Dielectric interface characterization for MoS_2 dual gated field effect transistor (DGFET).

Supplementary Figure. 1a and 1b show the dual sweep for the back-gate and top-gate transfer characteristics of MoS_2 DGFET with corresponding hysteresis windows (ΔV) of approximately 3 V and 2.5 V, respectively. Since hysteresis window is a measure for the degree of trapping at the 2D/gate-dielectric interface, it is fair to assume that the two interfaces are very similar in nature. Furthermore, similar values of constant-current threshold voltages were extracted from both forward ($V_{TH\text{-}forward}$) and reverse sweep ($V_{TH\text{-}reverse}$) of the back-gate and top-gate characteristics that reinforces our claim.

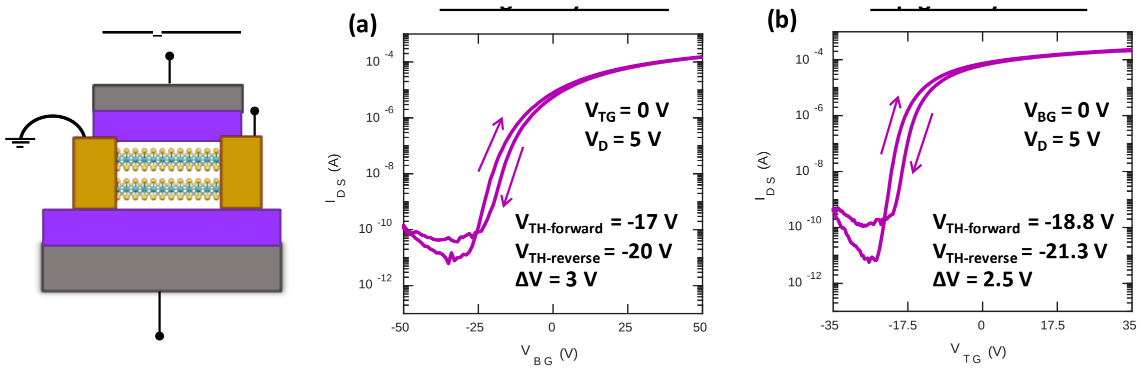

Supplementary Figure 1. Dielectric interface characterization for MoS_2 DGFET. *Dual sweep for a) back-gate and b) top-gate transfer characteristics of MoS_2 DGFET. The hysteresis windows (ΔV) are ~3 V and ~2.5 V, respectively. Since hysteresis window is a measure for the degree of trapping at the 2D/gate-dielectric interface, it is fair to assume that the two interfaces are very similar values of constant-current threshold voltages were extracted from both forward ($V_{TH\text{-}forward}$) and reverse sweep ($V_{TH\text{-}reverse}$) of the back-gate and top-gate characteristics.*

Supplementary Note 2: Design of Gaussian synapses

The dependence of amplitude (A), mean (μ_V), and standard deviation (σ_V) of the Gaussian synapse on the top gate voltages, V_N and V_P, derived using the physics based model in the main article can be empirically represented by the following equations:

$$\mu_V = \frac{V_N + V_P}{2}$$

$$\sigma_V \approx a_0 + a_1(V_P - V_N);\ a_0 = 0.04\,V;\ a_1 = 0.2\,V$$

$$A \approx a_2 \sigma_V V_{DD};\ a_2 = 12\ \mu A\ V^{-2}$$

Note that, a_0, a_1, and a_2 are constants, which are determined by the mobility of the respective 2D materials and the physical dimensions of the Gaussian synapse including the length, and

width of the n-type and p-type FET and the thicknesses of the gate oxides. These empirical equations are useful as these provide guide for circuit and architectural design and analysis of PNNs based on Gaussian synapses.

Supplementary Note 3: Calculation for Power Consumption by PNN

The static power consumption (P_{static}) by the proposed PNN architecture for brainwave detection was calculated based on the following equation:

$$P_{\text{static}} = \sum_{i=\delta,\theta,\alpha,\beta,\gamma} \sum_{j=1}^{N_{GAUSSIAN}} \sum_{n=1}^{N_{sample}} A_{I-i,j} \exp\left[-\frac{\{V_{\text{BG}}(n) - \mu_{v-i,j}\}^2}{2\sigma_{v-i,j}^{\ 2}}\right] V_{\text{DS}}(n)\,;$$

$$V_{\text{BG}}(n) = V_{\text{DD}} \frac{f(n)}{f_{\text{max}}};\ V_{\text{DS}}(n) = V_{\text{DD}} \frac{PSD(n)}{PSD_{\text{max}}}$$

In the above equation, $f(n)$ and $PSD(n)$ are, respectively, the nth frequency components and the corresponding power spectral density (PSD) of the post-FFT test sample data, and $V_{\text{BG}}(n)$ and $V_{\text{DS}}(n)$ are, respectively the corresponding scaled back-gate voltage and source to drain voltage applied to the PNN for a given supply voltage (V_{DD}).
